# Detection of Injury Biomarkers in Sweat of Collegiate Athletes Pre- and Post-Football Season: A Pilot Study

**DOI:** 10.1177/08977151251367345

**Published:** 2025-08-25

**Authors:** Sarah E. Svirsky, Christopher C. Wood, Olivia Raymond, Peyton McIntyre, Hannah Appleton, Chelsea Wagner, Jessica Gill, Ava M. Puccio

**Affiliations:** ^1^ Department of Neurological Surgery, University of Pittsburgh, Pittsburgh, Pennsylvania, USA.; ^2^ School of Nursing, Johns Hopkins University, Baltimore, Maryland, USA.

**Keywords:** glial fibrillary acidic protein, neurofilament light, sweat biomarkers, tau, traumatic brain injury, ubiquitin carboxyl-terminal hydrolase L1

## Abstract

The sports medicine community and society at large have recognized traumatic brain injury (TBI) as a major public health concern. It is estimated that more than 150 million youths have played football in the United States. As an alternative to blood, sweat is a potential source for protein biomarkers, providing a non-invasive method for objective measurements for head safety guidelines. This pilot study explores sweat as a means of detecting protein biomarkers of brain injury before and after a football season. Participants were football players from an NCAA Division III college (*N* = 34 pre-season, *N* = 18 post-season). At pre- and post-season time points, demographic, injury history, and physical activity assessments were conducted, including application of a non-invasive sweat patch for approximately 24 h. Sweat protein biomarkers total-tau, neurofilament light, glial fibrillary acidic protein, and ubiquitin carboxyl-terminal hydrolase L1 (UCH-L1) were measured via immunoarray. Paired and un-paired non-parametric statistical analyses were conducted. Athletes reported little to no concussion injuries from the season and experienced minimal symptoms. There was a significant increase in pre-season GFAP and UCH-L1 protein levels in athletes with a history of TBI compared to those without. Comparing between pre- and post-season, there was an increase in total-tau and UCH-L1 levels. These data suggest that sweat may be a viable biofluid to assess head injury using hallmark TBI biomarkers.

## Introduction 

A 2021–2022 report showed a record number of collegiate student-athletes in NCAA championship sports, surpassing 520,000 participants. The sports with the largest increase in participation in athletes include football (up 9%) and men’s and women’s soccer (up 11% and 7%, respectively), both with repetitive high-impact involvement.^1^ In conjunction, the sports medicine community and society at large now recognize concussion (i.e., mild traumatic brain injury, or mTBI) as a major public health concern. There are increasing efforts to refine not only guidelines for ‘return to play’ but also to identify objective measures to monitor a ‘tipping point’ of repeated damage for increasing long-term athletes’ health and safety.^2,3^ Particularly in the realm of sport-related concussion, fluid biomarkers have the potential to provide objective measures to support conventional diagnostic tools and track physiological recovery.^4–6^

While blood is the most common biofluid investigated for biomarkers, collection is invasive and requires well-trained personnel for proper collection and processing and specific facilities and/or equipment with analysis capabilities, which are not always locally accessible.^7^ In contrast, sweat is a potential strategy for non-invasive biomarker collection and is easier to access with minimal training. Sweat is a clear, odorless substance that consists of various electrolytes, proteins, and peptides representing multiple physiological systems.^8^ Although relatively under-studied in neurological disorders, analysis of sweat patches in studies of aging, depression, Schizophrenia, and Parkinson’s disease has demonstrated altered expression of cytokines, peptides, proteins, and metabolites compared to their respective control groups.^9–12^ There has yet to be a study that examines sweat content in a cohort with head injury exposure.

This pilot study aims to detect current hallmark TBI biomarkers, namely total-tau, neurofilament light (Nf-L), glial fibrillary acidic protein (GFAP), and ubiquitin carboxyl-terminal hydrolase L1 (UCH-L1) in sweat samples of collegiate football players pre- and post- in a single, competitive football season. These four central nervous system (CNS) proteins have previously been used to indicate TBI in a clinical setting and represent neuronal and astrocytic damage across time after injury.^4^ We predict these proteins will be detectable in sweat and will increase in concentration over the course of a football season as a reflection of repetitive mTBI exposure.

## Methods

### Participants

Football players from an NCAA Division III college who were rostered for the 2023 season were enrolled under an approved University of Pittsburgh IRB. Written, informed consent was obtained from the athlete participant prior to any research activities being performed. Participants completed self-reported case report forms for demographic data, injury history, medical history, medications, and physical activity. The Ohio State University TBI Identification Method and Rivermead Post Concussion Symptoms Questionnaire and were used to measure self-reported concussion history and symptoms during the pre-season visit. At post-season, participants completed and verified their personal information as well as season-specific details (i.e., season peripheral and concussion injury history, number of games played, and position played). If a concussion occurred, participants were asked to repeat these measures during the post-season session. All data were obtained via self-report measures. There were 34 participants in the pre-season (August, 1 week ahead of the season) and 18 returning participants in the post-season (December, 3 weeks after the season end).

### Sweat patch administration and sample collection

Sweat was collected using an individual sweat patch, which is a non-occlusive, hypoallergenic collection device, similar to a small adhesive bandage (PharmChem, Fort Worth, TX). Per manufacture instructions and as previously described,^12,13^ the placement of the patch was performed on the same day for all participants, within a 2-h window (Supplementary Fig. S1). The site was cleaned with alcohol, and the patch was applied on the upper arm in an area of skin that was free of hair. Emotionally induced sweating tends to be limited to the eccrine glands of the forehead, soles, and palms.^14^ Therefore, collecting sweat from the abdomen or flank helps to avoid capture of emotionally induced sweat excretion. Participants kept the patch on for approximately 24 h. Patch removal was performed on the following day for all participants. Each patch was labeled with the participant’s unique identifier along with the date and time of patch placement and removal. Immediately following removal, the patch was placed in a plastic bag, and within 1 minute, the patch was deposited in a cooler of dry ice for transport. Patches were stored in a −80°C freezer and subsequently shipped on dry ice to John’s Hopkins University (Baltimore, Maryland) and stored at −80°C until extraction. Two pre-season sweat patches were lost to follow-up.

### Sample processing

As previously described,^12^ the sweat patches had been stored at −80°C until analysis. For extraction and analysis, the sweat patches were thawed on ice. Each white absorption pad of the patch was removed using tweezers, avoiding contamination, and placed in a spin column of a 5 mL filtration centrifuge tube. Then, 500 μL of buffer consisted of 1× phosphate-buffered saline with 0.5% Tween20 and 0.2% bovine serum albumin was added to the spin column portion with the sweat pad, incubated at 4°C for 20 min, then centrifuged for 20 min at 4000 × *g* at 4°C. The filtered-through portion was collected and aliquoted to appropriately labeled sample tubes.

### Biomarker detection

Total-tau, Nf-L, GFAP, and UCH-L1 sweat concentrations were measured with an ultra-sensitive single-molecule enzyme-linked immunoarray commercially available Neurology 4-Plex A Advantage kit (Simoa,™ Quanterix Corporation, Lexington, MA), by a trained laboratory technician using a method previously described.^12^ This is a multiplex digital immunoassay for the simultaneous quantitative determination of low-abundance levels of all four proteins. All samples were run in duplicate. Samples were run with a HD-X instrument Quanterix at a 4x dilution. The lower limits of detection for tau, Nf-L, GFAP, and UCH-L1 were 0.024 pg/mL, 0.104 pg/mL, 0.221 pg/mL, and 1.74 pg/mL, respectively.

### Statistics

Descriptive statistics with means and proportions were used to describe categorical variables. Normality of biomarker distribution was tested using the Shapiro-Wilk test. Comparisons of pre-season biomarker concentrations between those with and without TBI history were analyzed using the Mann–Whitney test. Comparisons of paired pre-season and post-season biomarker concentrations were analyzed using the Wilcoxon matched-pairs signed-rank test. *p*-Values below 0.05 were considered significant. Data were analyzed using GraphPad Prism software (San Diego, CA).

## Results

### Athlete demographics and injury history

A total of 34 collegiate football players enrolled in the study during the pre-season (Table 1). TBI history was determined by the OSU-TBI self-reported questionnaire. At pre-season evaluation, 13 athletes reported a previous TBI, with 5 of those being a TBI with loss of consciousness. TBI-related symptoms were assessed by the RPQ and BSI. The median total sum score for the RPQ was 0 (range 0–3), and the median total sum score for the BSI was 0 (range 0–46). No concussions were reported during the football season of the enrolled participants. For the 18 returning athlete participants, the median number of football games played during the season was 4.5 out of a total of 10 games (range 0–10). Two participants did not play in a game during the season. At the pre-season time point, sweat patches were worn an average of 19.03 h. 6 of the 34 participants patches fell off or were accidentally removed prior to official patch collection the next day. 2 of the 34 patches were not returned. In the post-season, sweat patches were worn for an average of 26.92 h. All post-season patches remained adhered until official patch collection.

**Table 1.** Demographics, Injury History, Sweat Patch Application and Biomarker Levels at Pre- and Post-Season

	Pre-season	Post-season
Total (*N*)	34	18
Age (mean ± SD)	19.44 ± 1.50 years	19.56 ± 1.46
Range	18–23	18–22
Position played (*N*, %)		
Offensive/defensive line	8 (23.5%)	5 (27.8%)
Linebacker/quarterback/running back	14 (41.2%)	7 (38.9%)
Defensive back/tight end/wide receiver/special teams	10 (29.4%)	6 (33.3%)
Kicker/punter	2 (5.9%)	0 (0%)
OSU-TBI		
History of TBI Yes/No (*N*, %)	13 (38.2%)	n/a
TBI w/ LOC (*N*, %)	5 (14.7%)	n/a
Behavioral symptom testing		
RPQ median sum score (range)	0 (0–3)	n/a
BSI-18 median sum score (range)	0 (0–46)	n/a
Number of games played (median)	n/a	4.5
Range	n/a	0–10
Time patch worn (mean ± SD)	19.03 ± 8.1 h	26.92 ± 0.52 h
Range	2.75–24.25	25.0–27.55
Sweat biomarker conc. (mean ± SD)		
Total-tau (pg/mL)	0.89 ± 0.91	3.18 ± 1.94
Nf-L (pg/mL)	1.55 ± 3.14	2.79 ± 3.97
GFAP (pg/mL)	1.59 ± 0.90	7.52 ± 12.00
UCH-L1 (pg/mL)	40.74 ± 14.27	125.30 ± 165.10

BSI-18, Brief Symptom Inventory; Conc, Concentration; GFAP, glial fibrillary acidic protein; LOC, Loss of Consciousness; Nf-L, neurofilament light; OSU-TBI, Ohio State University TBI Identification Method; RPQ, Rivermead Post Concussion Symptoms Questionnaire; SD, Standard deviation; and UCH-L1, ubiquitin carboxyl-terminal hydrolase L1.

### Sweat biomarkers

**FIG. 1.**
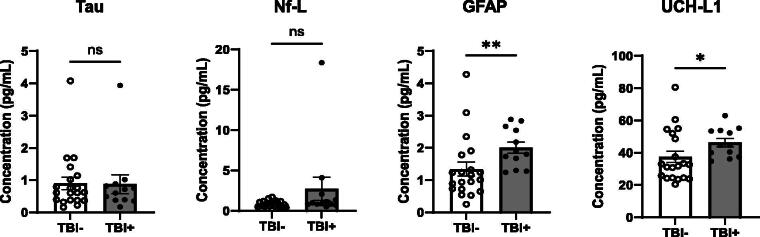
Pre-season, GFAP, and UCH-L1 are elevated in collegiate athletes with a self-reported history of TBI. Bar graphs show expression of total-tau, neurofilament light (Nf-L), glial fibrillary acidic protein (GFAP), and ubiquitin carboxyl-terminal hydrolase L1 (UCH-L1) in sweat between athletes without a history of TBI (TBI-, *n* = 19, open circles, white bar) and athletes who self-reported a previous TBI (TBI+, *n* = 13, closed circles, gray bar). Protein levels of un-paired samples were compared by the Mann–Whitney test. *p < 0.05, **p < 0.01, ns, non-significant.

**FIG. 2.**
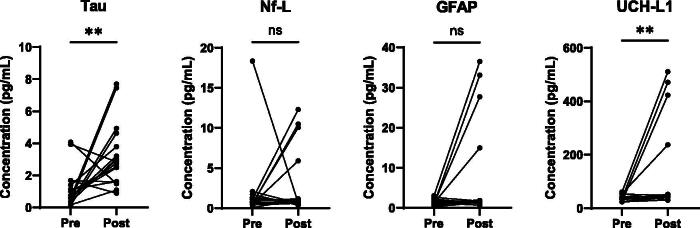
Total-tau and UCH-L1 are elevated in sweat of collegiate athletes from pre- and post-football season. Graphs show expression of total tau, neurofilament light (Nf-L), glial fibrillary acidic protein (GFAP), and ubiquitin carboxyl-terminal hydrolase L1 (UCH-L1) in sweat between pre-season (pre) and post-season (post) time points. Protein levels of paired samples (*N* = 17) were compared by the Wilcoxon matched-pairs signed-rank test. **p < 0.01, ns, non-significant.

Four hallmark injury biomarkers, Total-Tau, Nf-L, GFAP, and UCH-L1 were measured in sweat samples of college football players collected pre-season and post-season (Fig. 1). Shapiro-Wilk tests for normality showed that data across all four analytes measured did not meet criteria for normality (*p* < 0.05); therefore, Mann–Whitney test and Wilcoxon matched-pairs signed-rank tests were conducted to compare change in levels across un-paired and paired samples, respectively. Mann–Whitney un-paired statistical analysis (Fig. 1) showed a significant increase in GFAP and UCH-L1 levels in athletes with a history of TBI compared to those without (*p* = 0.0036 and *p* = 0.0169, respectively). There were no significant differences in total-tau or Nf-L. Paired analysis (Fig. 2) showed significant increases in total-tau and UCH-L1 levels between pre- and post-season (*p* = 0.0046 and *p* = 0.0032, respectively). There was no significant difference in Nf-L and GFAP levels between pre- and post-season (*p* = 0.6777 and *p* = 0.0638, respectively).

## Discussion

This is the first report, to our knowledge, to detect hallmark TBI proteins in sweat in addition to being the first study in contact sport athletes to examine sweat as a potential biomarker. Athletes reported little to no concussion injuries from the season and experienced minimal to no symptoms. However, sweat samples showed significant changes in protein levels based on TBI history as well as from pre- to post-season. This suggests that sweat biomarkers may be sensitive enough to detect previous exposure to TBI and participation in football season prior to behavioral symptom presentation.

While the use of blood biomarkers has advanced our understanding of the short- and long-term consequences of repetitive mTBI, its application within the contact sport realm is not as well understood. Temporal evaluations of serum and plasma in contact sports athletes at pre-season, post-season, or immediately post-injury show increases in GFAP, UCH-L1, Nf-L, and/or total-tau.^15–18^ In some cases, these increases occur independently of reported TBI events.^17^ We observed similar injury-dependent changes in sweat GFAP, UCH-L1, and injury-independent changes in total-tau in sweat. This speaks to the unique baseline physiology of the athlete population or a sensitivity of these biomarkers to detect a subconcussive injury paradigm. Future work aims at collecting blood concurrently with sweat to further our understanding of the utility of sweat as a matrix, not only as a reflection of blood biomarkers but as an independent indicator of repetitive mTBI pathophysiology.

Beyond these four CNS-focused biomarkers, inflammatory cytokines appear to be lucrative targets for detection of disease states in sweat. IL-1α, IL-1β, IL-6, IL-8, IL-10, and TNFα levels are increased in sweat in cohorts of patients across various disease states and conditions. These observations reflect concurrent with increases of pro-inflammatory cytokines in the blood.^9,13^ Similarly, mild TBI triggers a neuroinflammatory response, with elevated blood levels of the same cytokine profile in the days following injury.^19^ Future studies could investigate the performance of inflammatory markers as a biomarker of TBI in sweat.

There are several limitations to this study. First, there was no non-impact comparison group nor female athletes included. To determine the specificity of our observations to head impacts, comparisons of sweat content should be made to, preferably, a non-contact sport athlete, where similar cardiovascular exercise is performed. To evaluate sex differences, a female athlete cohort should be included. Second, there is no standardized method to normalize sweat collection and total sweat content. While the time sweat patch is adhered to and extraction of sweat samples is relatively controlled, there is expected to be variability across participants in the volume and rate of sweat and total content. Sweat mass, or sweat volume, has previously been the primary method for standardizing the sweat rate for biomarker evaluation. However, given that sweat is approximately 99% water, measurement of sweat ion concentration is believed to be a more accurate method of standardization.^20,21^ Particularly relevant to the athlete population, rate-normalized features can also be correlated to physical performance and fitness metrics.^21^ Tailoring standardization methods to the patient population of interest is crucial to optimizing sweat biomarker methods and implementation in appropriate settings.

## Conclusion

This pilot study successfully measured protein levels of total-tau, Nf-L, GFAP, and UCH-L1 in sweat samples of collegiate football players and detected select increases in levels based on TBI history and over the course of a single football season. These data suggest that sweat may be a viable biofluid to assess hallmark TBI biomarkers.

## Transparency, Rigor, and Reproducibility

This study was not formally registered because it is a preliminary investigation. A sample size of 34 subjects was planned based on the availability of volunteers from an NCAA Division III football program. Thirty-four participants had sweat patches administered at the pre-season time point. Two sweat patches were lost to follow-up and were not collected for analysis. Thirty-two participant sweat patches were analyzed. Eighteen participants had sweat patches administered at the post-season time point and all 18 sweat patches were analyzed. All 34 participants had initial clinical and demographic outcomes assessed. Participants were blinded to the results of the biomarker measurements. Quality control decisions and analyses were performed by investigators blinded to characteristics of the participants. Sweat patches were labeled using unique codes that were not linked to participant identifying information. Samples were collected at the college campus using sweat patches and stored on dry ice or in a −80 freezer until analysis. All samples were analyzed at the same time in a single batch.

Samples were diluted 4 times using sample diluent, per instructions of the Quanterix Simoa Neurology 4-plex A Advantage Kit (item number 102153). The number of technical replicates was 2 per sample. The inter-run and inter-instrument coefficient of variation for each of the proteins was less than 11%. All equipment and analytical reagents used to perform measurements are widely available from Pharmchek (sweat patch) and Quanterix (assay). The statistical tests used were based on the assumptions of nonnormality. Replication by the study group is ongoing. De-identified data from this study are not available in a public archive.

## Abbreviations Used

BSI: Brief Symptom Inventory
CNS: Central nervous system
conc: Concentration
GFAP: Glial fibrillary acidic protein
IL: Interleukin
LOC: Loss of Consciousness
mTBI: Mild TBI
NfL: Neurofilament light
OSU-TBI: Ohio State University TBI Identification Method
RPQ: Rivermead Post-Concussion Symptoms Questionnaire
SD: Standard deviation
TBI: Traumatic brain injury
TNF: Tumor necrosis factor
UCH-L1: Ubiquitin carboxyl-terminal hydrolase L1

## References

[1] NCAA. NCAA Sports Sponsorship and Participation Rates Database - NCAA.org [Internet]. NCAA.org. 2024 [cited 2025 Jan 8]. Available from: https://www.ncaa.org/sports/2018/10/10/ncaa-sports-sponsorship-and-participation-rates-database.aspx

[2] May KH, Marshall DL, Burns TG, et al. Pediatric sports specific return to play guidelines following concussion. Int J Sports Phys Ther 2014;9(2):242–255. 24790785 PMC4004129

[3] Kamins J, Bigler E, Covassin T, et al. What is the physiological time to recovery after concussion? A systematic review. Br J Sports Med 2017;51(12):935–940. 28455363 10.1136/bjsports-2016-097464

[4] Senaratne N, Hunt A, Sotsman E, et al. Biomarkers to aid the return to play decision following sports-related concussion: A systematic review. J Concussion 2022;6:205970022110707.

[5] Tabor JB, Brett BL, Nelson L, et al. Role of biomarkers and emerging technologies in defining and assessing neurobiological recovery after sport-related concussion: A systematic review. Br J Sports Med 2023;57(12):789–797. 37316184 10.1136/bjsports-2022-106680

[6] McCrory P, Meeuwisse W, Dvořák J, et al. Consensus statement on concussion in sport BMJ 2017. Br J Sports Med 2017;51(11):838–847. 28446457 10.1136/bjsports-2017-097699

[7] Tuck MK, Chan DW, Chia D, et al. Standard operating procedures for serum and plasma collection: Early detection research network consensus statement standard operating procedure integration working group. J Proteome Res 2009;8(1):113–117. 19072545 10.1021/pr800545q PMC2655764

[8] Burat B, Reynaerts A, Baiwir D, et al. Characterization of the human eccrine sweat proteome-A focus on the biological variability of individual sweat protein profiles. Int J Mol Sci 2021;22(19):10871. 34639210 10.3390/ijms221910871 PMC8509809

[9] Cizza G, Marques AH, Eskandari F, et al.; POWER Study Group. Elevated neuroimmune biomarkers in sweat patches and plasma of premenopausal women with major depressive disorder in remission: The POWER study. Biol Psychiatry 2008;64(10):907–911. 18657799 10.1016/j.biopsych.2008.05.035 PMC2610843

[10] Raiszadeh MM, Ross MM, Russo PS, et al. Proteomic analysis of eccrine sweat: Implications for the discovery of schizophrenia biomarker proteins. J Proteome Res 2012;11(4):2127–2139. 22256890 10.1021/pr2007957 PMC3703649

[11] Hirayama M, Tsunoda M, Yamamoto M, et al. Serum Tyrosine-to-Phenylalanine ratio is low in Parkinson’s disease. J Parkinsons Dis 2016;6(2):423–431. 27061063 10.3233/JPD-150736

[12] Hladek MD, Szanton SL, Cho Y-E, et al. Using sweat to measure cytokines in older adults compared to younger adults: A pilot study. J Immunol Methods 2018;454:1–5. 29128425 10.1016/j.jim.2017.11.003 PMC5818291

[13] Marques-Deak A, Cizza G, Eskandari F, et al.; Premenopausal, Osteoporosis Women, Alendronate, Depression Study Group. Measurement of cytokines in sweat patches and plasma in healthy women: Validation in a controlled study. J Immunol Methods 2006;315(1–2):99–109. 16942779 10.1016/j.jim.2006.07.011

[14] Sato K, Kang WH, Saga K, et al. Biology of sweat glands and their disorders. I. Normal sweat gland function. J Am Acad Dermatol 1989;20(4):537–563. 2654204 10.1016/s0190-9622(89)70063-3

[15] Gill J, Merchant-Borna K, Jeromin A, et al. Acute plasma tau relates to prolonged return to play after concussion. Neurology 2017;88(6):595–602. 28062722 10.1212/WNL.0000000000003587 PMC5304458

[16] McCrea M, Broglio SP, McAllister TW, et al.; CARE Consortium Investigators. Association of blood biomarkers with acute sport-related concussion in collegiate athletes: Findings from the NCAA and department of defense CARE consortium. JAMA Netw Open 2020;3(1):e1919771. 31977061 10.1001/jamanetworkopen.2019.19771 PMC6991302

[17] Laverse E, Guo T, Zimmerman K, et al. Plasma glial fibrillary acidic protein and neurofilament light chain, but not tau, are biomarkers of sports-related mild traumatic brain injury. Brain Commun 2020;2(2):fcaa137. 33543129 10.1093/braincomms/fcaa137 PMC7846133

[18] Shahim P, Tegner Y, Marklund N, et al. Neurofilament light and tau as blood biomarkers for sports-related concussion. Neurology 2018;90(20):e1780–e1788. 29653990 10.1212/WNL.0000000000005518 PMC5957307

[19] Malik S, Alnaji O, Malik M, et al. Inflammatory cytokines associated with mild traumatic brain injury and clinical outcomes: A systematic review and meta-analysis. Front Neurol 2023;14:1123407. 37251220 10.3389/fneur.2023.1123407 PMC10213278

[20] Hussain JN, Mantri N, Cohen MM. Working up a good sweat - The challenges of standardising sweat collection for metabolomics analysis. Clin Biochem Rev 2017 Feb;38(1):13–34. 28798503 PMC5548369

[21] Harshman SW, Strayer KE, Davidson CN, et al. Rate normalization for sweat metabolomics biomarker discovery. Talanta 2021;223(Pt 1):121797. 33303130 10.1016/j.talanta.2020.121797

