# Supplementary material for: Detection of Injury Biomarkers in Sweat of Collegiate Athletes Pre- and Post-Football Season: A Pilot Study
Source: Neurotrauma Rep. 2025 Aug 25;6(1):700–5. doi: 10.1177/08977151251367345 (PMC12413254; doi:10.1177/08977151251367345)
Supplement: Supplementary Figure S1 [file 08977151251367345_supplementary_figure_s1.docx]

**
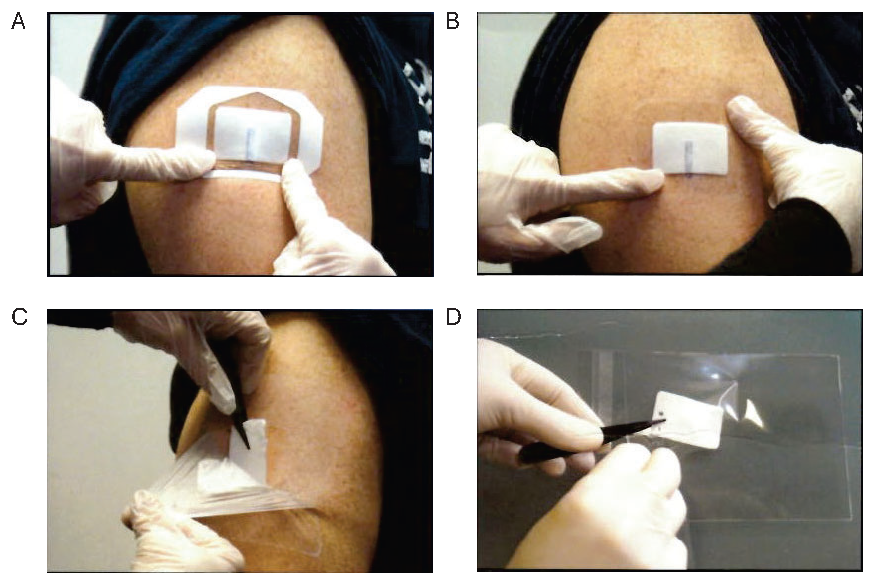
**

**Supplementary Figure 1:** Application and Removal of Sweat Patch. Before applying the sweat patch, the application area is cleaned with an alcohol wipe. (A-B) The sweat patch is applied and the absorbent pad is held in place with a protective adhesive polyurethane film. (C-D) Approximately 24 hours later, the film is removed and using forceps, the patch is placed directly in a biohazard bag for immediate storage at -80°C until analysis.
